# Probing the Structural Order of Half-Heusler Phases in Sb-Doped (Ti,Zr,Hf)NiSn Thermoelectrics

**DOI:** 10.3390/nano15131037

**Published:** 2025-07-03

**Authors:** Fani Pinakidou, Andreas Delimitis, Maria Katsikini

**Affiliations:** Department of Physics, Aristotle University of Thessaloniki, GR-54124 Thessaloniki, Greece

**Keywords:** Half-Heusler (HH), thermoelectrics (TEs), nanostructure, Extended X-ray Absorption Fine Structure Spectroscopy (EXAFS), Transmission Electron Microscopy (TEM/HRTEM), Energy Dispersive X-ray Spectroscopy (EDS)

## Abstract

The nanostructural features of a mechanically alloyed Sb-doped (Ti_0.4_Zr_0.6_)_0.7_Hf_0.3_NiSn thermoelectric (TE) Half-Heusler (HH) compound were addressed using Transmission Electron Microscopy (TEM) coupled with Energy Dispersive Spectroscopy measurements and Extended X-ray Absorption Fine Structure (EXAFS) spectroscopy. The EXAFS measurements at the Ni-*K*, Sn-*K,* Zr-*K,* and Hf-*L*_3_-edge were implemented in an effort to reveal the influence of Hf and Zr incorporation into the crystal with respect to their previously measured TE properties. The substitution of Ti by Hf and Zr is expected to yield local lattice distortions due to the different atomic sizes of the dopants or/and electronic charge redistribution amongst the cations. However, the material is characterised by a high degree of crystallinity in both the short and long-range order, on average, and the nominal stoichiometry is identified as (Zr_0.42_Hf_0.30_Ti_0.28_)NiSn_0.98_Sb_0.02_. The synergistic effect of minimization of extended structural defects or lattice distortions and considerable alloying-induced point defect population contributes to the improved TE properties and leads to the previously reported enhancement of the figure of merit of the mixed HHs.

## 1. Introduction

Half-Heusler (HH) compounds are a class of intermetallic materials that belong to the broader category of Heusler alloys. They are particularly known for their unique combination of properties, which render them promising materials for a range of applications, including thermoelectrics (TEs), magnetic materials, and shape memory alloys [1,2,3]. This group of compounds is at the forefront of alternative energy harvesting technologies, specifically in thermoelectric power generation [4,5], since they enable energy harvesting from waste heat in industries, vehicles, and even from natural sources like geothermal energy [6,7]. By improving the efficiency of energy conversion from waste heat, HH materials hold significant potential to reduce greenhouse gas emissions and enhance the sustainability of power systems [8].

In recent years, HH compounds have gained significant attention as promising TE materials due to their excellent balance between electrical conductivity, thermal conductivity, and Seebeck coefficient. In particular, they combine a high Power Factor (PF) with low thermal conductivity and a high thermoelectric figure of merit (*zT*) [9,10]. The latter can be achieved by combining increased values of the Seebeck coefficient and a low thermal conductivity. Furthermore, the electronic (κ_el_) and phonon (κ_lat_) components, attributed to mobile charge carriers and lattice vibrations, respectively, have a strong impact on the thermal conductivity of the compound. Among several types of ΤΕ materials developed over the past few years, HH compound elements are non-toxic and can be found in high abundance in nature; compared to commercially used TE elements (e.g., Te, Pb), they are also cost-effective [11,12]. Additionally, their application can be realized in a wide temperature range (from medium to high) while their high thermal and mechanical reliability renders them as strong candidates for TE generators (TEGs) [13]. Yet, they possess a relatively high lattice thermal conductivity κ_lat_ [14,15], consequently restricting, to a significant degree, their thermoelectric performance. Ways to accommodate this includes alloying and nanostructuring approaches, in an effort to increase phonon scattering through mass fluctuations, creation of a significant amount of grain boundaries and imposed strain, i.e., n-type MNiSn and p-type MCoSb with M = Ti, Zr or Hf [16,17], Sb-doped Hf_0.25_Zr_0.25_Ti_0.5_NiSn [18], and other p-type HH compounds [19]. In particular, for n-type HHs, phase homogeneity and carrier control by fine-tuned doping during synthesis are critical issues, as they impose further constraints compared to p-type materials. Sb acts as a promising dopant in n-type HHs, providing a high electron concentration and, hence, decreasing electrical resistivity [16,18,20,21]. In addition, Sb often substitutes for Sn sites, thus creating new scattering centers (point defects), which effectively scatter low-frequency phonons and decrease thermal conductivity. The synergistic effect of boosting donor concentration and reducing thermal conductivity [21] results in a large increase in *zT*, rendering Sb doping among the most promising ways for TE property improvement.

Previous studies highlighted the necessity of introducing lattice point defects [22,23], i.e., interstitials and/or substitutional ions, as an effective way of scattering the short-wavelength phonons, thus reducing lattice thermal conductivity. However, the exact nature, type, and location of such defect centers have yet to be clarified in order to effectively design n-type HHs with enhanced TE properties, i.e., low thermal conductivity, combined with a high Seebeck coefficient, power factor, and *zT*.

**Figure 1:**
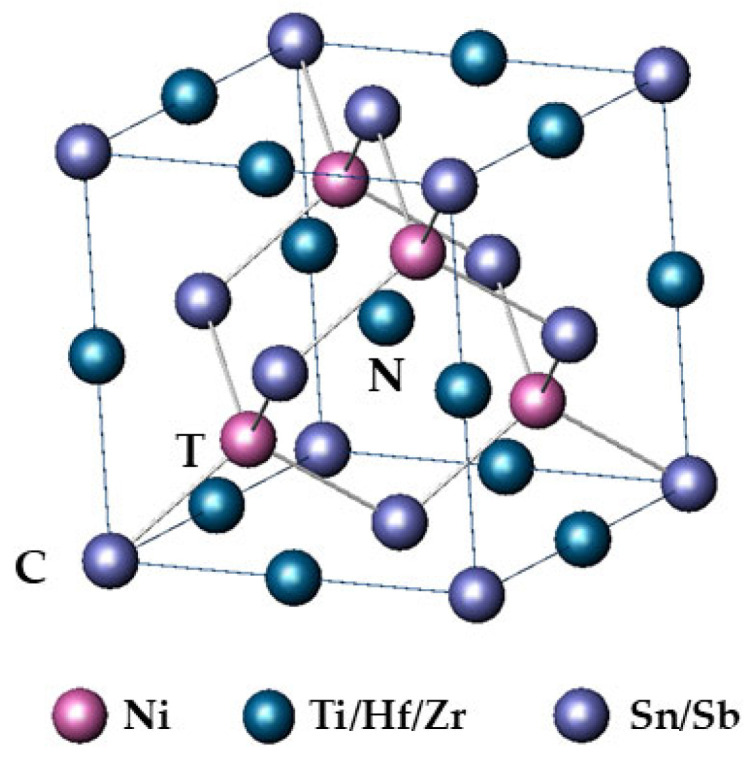
Model crystal structure of a Half-Heusler compound; T represents the tetrahedral 4c sites and C and N the 4a and 4b octahedral sites, respectively.

TiNiSn-based HH compounds crystallize at the high symmetry F$\bar{4}$3 m space group (#216), i.e., zinc blende, comprising three interpenetrating fcc sublattices. In principle, in an ideal unit cell, Sn ions occupy the octahedral 4a (0,0,0) sites (C sites), Ti atoms occupy octahedral 4b (½, ½, ½) sites (N sites), whereas Ni ions occupy one of the two distinct tetrahedral 4c (¼, ¼, ¼) or (¼, ¼, ¾) sites (T sites) (Figure 1). The other tetrahedral site remains, in general, unoccupied in HH compounds, whereas it is filled in full Heusler compounds. Upon doping or alloying, the additional ions generally occupy the respective sites of the substitutional ion; nevertheless, occupancy in other sites has often been reported [24].

This study addresses the issue of identifying the type, site, and percentage of host and dopant ions in a Sb-doped (Zr,Hf,Ti)NiSn HH alloy by a combination of Transmission Electron Microscopy (TEM) methods—conventional and High Resolution imaging (TEM and HRTEM), Energy Dispersive X-ray Spectroscopy (EDS)—and Extended X-ray Absorption Fine Structure (EXAFS) Spectroscopy. The samples were prepared by mechanical alloying (MA), i.e., ball milling, as an inexpensive, timely, and scalable method of synthesizing advanced TE nanomaterials. In particular, only a few studies have reported the successful growth of solid solutions of n-type HHs, as such attempts are limited to their end members. The stoichiometry of the compound was identified via EDS, and the presence of local structural distortions induced by Hf and Zr alloying and Sb doping is addressed using HRTEM/EDS and EXAFS measurements at the Ni-*K*, Sn-*K*, Zr-*K* and Hf-*L*_3_ edges. More specifically, due to its element-specific character, EXAFS spectroscopy is sensitive to bond length variations since it probes the short-range order around a selected absorbing atom. Static disorder can also be identified by quantifying the mean square deviation in atomic positions, as this is determined by the Debye–Waller (DW) factors. Since cation/anion site vacancies and antisites, as well as chemical disorder (random occupation of crystallographic sites), are common in HH compounds, EXAFS is capable of detecting vacancies or displaced atoms by analyzing changes in the type of the coordination environment and bonding geometry, while it can easily distinguish between homogeneous alloys and materials with secondary phases.

## 2. Materials and Methods

High-purity elemental powders of Ti (99.99% Alfa Johnson Matthey GmbH, Sulzbach, Germany), Hf, Zr (99% US Research Nanomaterials Inc., Research Nanomaterials Inc, TX, USA), Ni (99.99% Sigma Aldrich Merck, Darmstadt, Germany), Sn (99.85% Alfa Johnson Matthey GmbH, Sulzbach, Germany), and Sb (99.9% Alfa Johnson Matthey GmbH, Sulzbach, Germany) were weighed according to the selected compositions and milled in a tungsten carbide ball milling vial at a speed of 600 rpm under Ar for an MA duration of between 6 and 12 h. The synthesis is described in more detail in [24]. Due to the most promising TE property measurement results and the degree of alloying, a sample containing all Ti, Zr, Hf, and Sb dopants has been selected for the X-ray absorption and electron microscopy experiments.

Samples suitable for nanostructural analysis at the TEM were prepared by dissolving the finely crushed material in high-purity ethanol and evenly dispersing it on ultrathin lacey C-films supported on 3.05 mm copper grids. TEM/HRTEM experiments were carried out in a JEOL JEM2100 and a JEOL JEM2010 TEM, both equipped with LaB_6_ electron sources and operating at 200 kV; their point resolutions are 0.25 nm and 0.194 nm, respectively. The EDS measurements were performed at the JEOL JEM2100 TEM equipped with an EDAX Apollo XLT silicon drift EDS detector having an ultra-thin window and an energy resolution of 129 eV (Mn-*K*). Spectrum acquisition and analysis were realized using the TEAM EDS Suite v2.0 software.

The EXAFS spectra of the studied HH compound were measured at the Ni-*K*, Sn-*K*, Zr-*K,* and Hf-*L*_3_ edges at the BM31 Swiss Norwegian beamline of the European Synchrotron Radiation Facility (ESRF). The beamline is equipped with a Si(111) double-crystal monochromator that covers a wide spectral range (4–90 keV). All spectra were recorded at room temperature in the transmission mode using a set of ionization chambers. The first detector is positioned in front of the sample and records the intensity of the impinging X-rays to the sample’s surface (I_0_); the second is placed after the sample in order to detect the transmitted beam. Data reduction involved the following processing steps: normalization of the raw data to the beam current I_0_, subtraction of the atomic background, and transformation from the energy- to the *k*-space [25]. Data analysis of the EXAFS data proceeded using proper photoelectron scattering paths built with the FEFF8 code [26], while the fitting was performed using FEEFIT [27].

## 3. Results and Discussion

**Figure 2:**
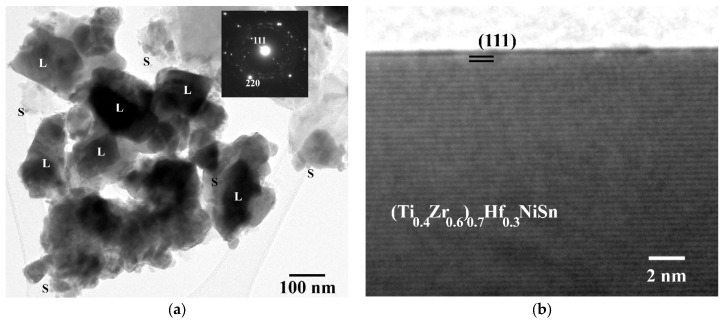
(**a**) Conventional TEM image of the particle categories in the HH sample, along with the SAD pattern of a typical large particle (inset); the letters “L” and “S” stand for large particles and nanoparticles, respectively. (**b**) HRTEM image from the edge of a large particle, where the (111) planes of the Sb-doped (Zr,Hf,Ti)NiSn mixed phase are revealed.

The detailed structural features of the HH material were elucidated by TEM/HRTEM experiments, combined with EDS analysis. In line with previously reported findings for Hf-free HHs [16], the particles in the studied sample can be grouped in two major categories, size-wise: relatively large particles, about 50–190 nm on average, and smaller, nanoscale particles of up to 15 nm in size. Both of these categories comprise single crystalline particles. The two distinct particle categories are illustrated in the typical image of Figure 2a, where the letter “L” is assigned to the large particles and “S” to the nanoparticles, respectively. A representative Selected Area Diffraction (SAD) pattern is also included (inset in Figure 2a). The HRTEM image in Figure 2b focuses on the edge of a typical large crystalline particle, where the (111) lattice planes are predominantly revealed. Measurements of the interplanar (111) plane spacings resulted in *d_111_* = 3.54 Å and a lattice constant of *a_HRTEM_* = 6.13 Å. For comparison reasons, the theoretical lattice parameter of the ternary (Zr,Hf,Ti)NiSn alloy has been determined by Vegard’s law, taking into account the ternary HHs TiNiSn (*a* = 5.94 Å), HfNiSn (*a* = 6.09 Å), and ZrNiSn (*a* = 6.12 Å), and their respective contents in the studied sample. This yields a lattice constant of *a_theor_* = 6.06 Å. Therefore, the large particle in Figure 2b exhibits a lattice constant larger by 1.15%, which implies an increased content of Hf and Zr compared to the nominal composition. In addition, measurements of the main and additional reflections at the SAD pattern (inset of Figure 2a) provided the values listed in Table 1.

The resulting lattice constants *a* from the SAD measurements are also in good agreement with the theoretical lattice parameter *a_theor_*. However, small variations of *a* are observed, i.e., the minimum and maximum values detected are −0.5% and 0.7%, respectively. Although such variations are small, they are well within the range for the mixed HH sample and its end members, especially HfNiSn. This finding can be attributed to mass fluctuations of the various host and Sb dopant ions in the HH lattice.

**Figure 3:**
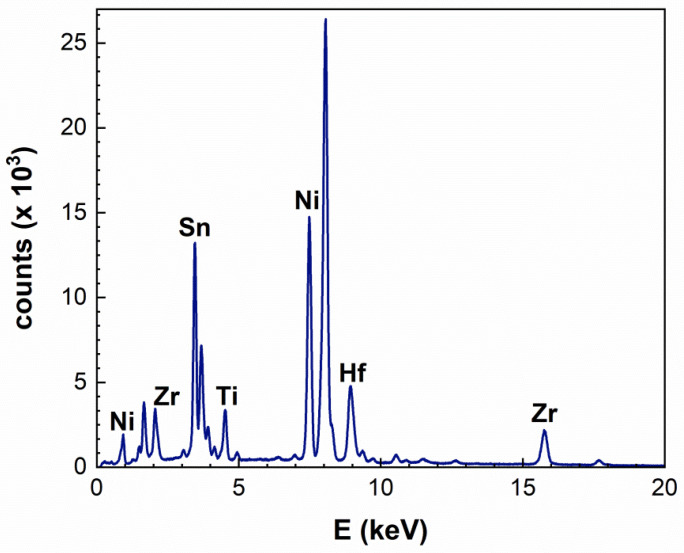
EDS spectrum from a large HH particle, showing the main elemental peaks and their average concentration. The peak at approximately 8 KeV stems from the Cu grid used for the TEM sample preparation.

In addition, EDS spectroscopy confirmed the aforementioned findings. In Figure 3, a typical EDS spectrum is illustrated, originating from a larger particle in the sample. The peaks of the main HH elements (Ti, Zr, Hf, Ni, Sn) are easily distinguished, whereas Sb was difficult to detect, both due to its low content and also to the overlapping with the Sn *L* family lines; therefore, the sum of these two elements will be considered in any quantitative calculations from now on. Quantitative analysis of the EDS spectra from all regions in the TEM sample has been performed, and the results are listed in Table 2. Therefore, it is confirmed that, in general, the elemental ratios are in good agreement with their theoretical values in the vast majority of the particles analysed. Zr and Hf showed some elemental fluctuations, with an opposite trend, which implies elemental inhomogeneities at the nanometer/atomic scales. This is also further confirmed by the fact that Hf-rich or Sn-rich particles have been detected by EDS; nevertheless, such particles constitute a rather small portion compared to the roughly stoichiometric ones. As SAD, HRTEM, and EDS analyses resulted in slight variations in stoichiometry among different crystallites, the material has been further investigated using EXAFS spectroscopy in order to derive complementary detailed information on the material’s nanostructure. Indeed, the EXAFS technique probes a larger volume of the alloy and can consequently describe the average of the local environment of the host/dopant atom. Overall, however, no phase separation [19] has been detected by SAD and HRTEM measurements, while it was confirmed by EDS that the particles, large or small, comprise a ternary mixed Sb-doped (Zr,Hf,Ti)NiSn phase.

A detailed investigation on the thermal properties in a series of (Ti_0.4_Zr_0.6_)_0.7_Hf_0.3_NiSn, where the Sb dopant substituted the Sn site with 1 at%, 1.5 at%, 2 at%, and 2.5 at%, was previously conducted by Mesaritis et al. [24]. In their work, a decrease in the Seebeck coefficient was reported, which was attributed to the increase in carriers induced by Sb incorporation; this finding was accompanied by an increase in both the electrical conductivity and power factor. Nevertheless, the incorporation of antimony into the lattice also led to a reasonable increase in the total thermal conductivity, following the respective increase in electrical conductivity. Yet, the figure of merit reached a value which was the best reported among the studied alloys, and, remarkably, both undoped and doped members experienced similar *zT*s that actually originate from opposite ranges of power factors and thermal conductivity.

**Figure 4:**
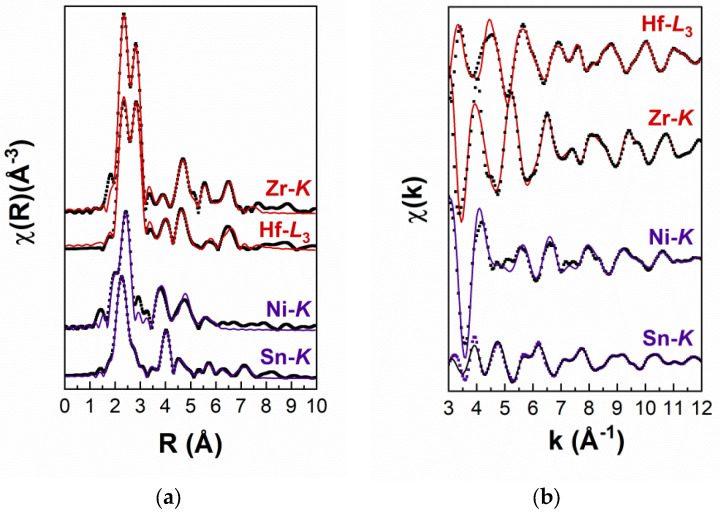
(**a**) Fourier transforms (FTs) of the *k*^3^-weighted χ(*k*) EXAFS spectra recorded at the *K*-edges of Sn, Ni, and Zr and *L*_3_-edge of Hf. (**b**) Raw and fitted χ(*k*) EXAFS spectra recorded at the *K*-edges of Sn, Ni, and Zr and *L*_3_-edge of Hf. The solid squares depict the experimental data, while the fitting is represented by a solid line.

In order to correlate the Zr/Hf alloying- and Sb doping-induced structural changes with the exceptional TE properties of the sample under investigation, EXAFS spectroscopy is applied as a more average and complementary technique to HRTEM/EDS. In particular, due to its atom-selective character, it can yield evidence on the bonding environment of the atoms of interest, as this is determined by the type and distance of the nearest neighbours and thermal and static disorder (Debye Waller factors). Thus, in order to extract information on the influence of Zr and Hf incorporation in the studied HH alloy, the fitting at the Ni-*K* edge and Sn-*K*, Zr-*K*, Hf-*L*_3_ edges was performed in the first seven and ten nearest neighbouring (nn) shells, respectively; i.e., at a distance from the absorbing atom of up to approximately 6.0 and 7.8 Å. The compound under investigation was modelled using the crystal structural model of cubic TiNiSn [28], where Ti and Sn occupy octahedral sites and Ni is located in tetrahedral sites. In order to take into account the random population of available Ti sites by the Hf and Zr atoms, the model was modified accordingly by randomly replacing 2/3 of the Ti atoms with equal amounts of Hf and Zr atoms. In particular, it was assumed that the stoichiometry of the studied compound is described by the formula (Zr_x_Hf_y_Ti_z_)NiSn_0.98_Sb_0.02,_ where x, y, and z are the atomic percentages (at%) of Zr, Hf, and Ti, respectively. According to the most typical EDS analysis results, the following constraints must apply: the at% of Zr is expected to be 1.4 times the at% Hf, i.e., x = 1.4 × y, and, also, x + y + z = 1 must hold. For all edges, the amplitude reduction factor was kept fixed to the value 0.9; additionally, the energy origin was iterated, and this is also the case for the values of the DW factors, which, however, were constrained to be equal among shells that contain the same type of atoms. The fitting was performed at the following *k*-ranges: 3.5–11.5 Å^−1^ (Ni-*K*), 3.0–14.0 Å^−1^ (Sn-*K*), and 3.5–13.5 Å^−1^ (Zr-*K* and Hf-*L*_3_) simultaneously at the *k*- and *R*-space. The raw and fitted EXAFS spectra in the *R*-space are shown in Figure 4a, and those in the *k*-space are shown in Figure 4b. The fitting results are listed in Table 3 (the errors listed are those determined by FEFFIT).

The fact that the Zr and Hf atoms do not appear, in general, to substitute for either Ni or Sn in the sample volume probed by EXAFS is demonstrated by the similarities and also differences among the profiles of the Fourier transforms (FTs) of the EXAFS spectra. More specifically, a splitting in the first nn shell (1st peak in the FTs) arises due to the partial substitution of Ti by Hf or Zr and is detected only in the case of the Hf-*L*_3_ and Zr-*K* edges in Figure 4a. This is not the case, however, for the FTs of the Ni-*K* and Sn-*K* EXAFS spectra, indicating the different bonding environment around the elements in question.

Indeed, as listed in Table 3, the EXAFS analysis results reveal that, on average, little substitution of both Ni and Sn sites exists. Instead, the Zr and Hf atoms preferentially occupy available octahedral Ti sites in the unit shell. The construction of the theoretical model used in the fitting process takes into account the lattice parameters obtained previously by Vegard’s law. According to this modified model, the Ni-Sn interatomic distance is expected to be equal to 2.62 Å. From the EXAFS analysis at the Sn- and Ni-*K* edges, it is revealed that the Ni-Sn bond length is practically the same (within the error bar) and equal to 2.60 and 2.61 Å (±0.01), respectively; i.e., the detected slight shift is within the limits determined by the nominal stoichiometry. This result lies in good agreement with the TEM/SAD analysis results, where the lattice parameter deviations in the majority of particles have been found within the theoretical value limits. Furthermore, in the second nn shell, the Ti atoms are expected at different distances around Ni and Sn: for the Ni sites, Ti is located at 2.64 Å (as in the case of the Sn atoms) and at a longer distance, i.e., 3.03 Å, from the Sn atoms. Again, no significant disorder is detected in this shell: the distance of Ti from both Ni and Sn remains very close (within the limits of the error bar) to the theoretically expected value. More specifically, following their partial substitution by Hf and Zr, the Ti atoms are located at 2.61 and 3.05 Å (±0.03) from the Ni and Sn atoms, respectively. Furthermore, the random distribution of Zr and Hf on the specific lattice site of Ti induces no local distortion (considering the determined error bars): in the vicinity of Sn, both types of atoms are found at 3.05 (±0.03) and 3.04 Å (±0.02), respectively, and at 2.60 Å (±0.03) from Ni. Moving to the longer range order, the Sn-Sn and Ni-Ni distances in the third nn shell are not significantly affected by the presence of Hf and Zr. Although the error in such distant nn shells is relatively large (±0.05), Sn and Ni atoms are detected at the same distance (4.27 Å).

As far as the DW factors are concerned, they are approximately equal to 6.0–6.5 × 10^−3^ Å^2^ for the first neighbors, [Sn-Ni] and [Ni-Zr/Hf], and slightly change in the second nearest neighbors, [Sn-Zr/Hf/Ti] and [Ni-Zr/Hf/Ti], depending on the type of atom. Previous reported XAFS work on a HH ZrNiSn alloy revealed the presence of atomic defects at the vacancy sites that lead to the distortion of the crystal structure, a finding that partially elucidated the material’s poor thermal behavior as compared to the theoretically predicted for HH alloys [29]. On the contrary, in another group of HH phases of the type (Ti,Zr,Hf)CoSb, the low thermal conductivity was attributed to an intrinsic phase separation in two HH phases, where Ti and Hf are distributed randomly on their common crystallographic lattice site without any exchange between different atoms or occupation of vacant lattice sites [30]. A theoretical approach to the thermoelectric properties of popular thermoelectric materials using EXAFS calculations revealed interesting data that confirm the indirect relation of thermoelectric properties with the decrease in the number of outer-shell electrons of the metals [31]. Nevertheless, in the studied HH compound, the population of Ti atoms by Hf and Zr does not play a crucial role in the material’s degree of crystallinity in the long-range order; the occupation of octahedral sites by the dopants does not yield considerable local distortions around Ni and Sn. In addition, TEM characterization proved the presence of one mixed HH phase in the sample, with no phase separation effects.

With regard to the environment around the dopants, the EXAFS analysis further supports the previous results. It is demonstrated that the Hf-Ni and Zr-Ni bond lengths are equal (within the error bar) to 2.61 and 2.64 Å (±0.01), i.e., as in the case of the Ni-*K* edge fitting results (2.63 ± 0.03 and 2.65 ± 0.02 Å). The Sn atoms in the 2nd nn shell are located at the same distance (3.05–3.03 Å (±0.02)) from each dopant (again within the error bar), in agreement with the respective distances found from the analysis at the Sn-*K* edge (3.05 ± 0.02–3.04 ± 0.03 Å). In the 3rd nn shell, the Zr-Hf and Zr/Hf-Ti distances are found equal to 4.28–4.27 Å (±0.04–0.05) and 4.27–4.30 Å (± 0.05), signifying, yet again, that the material possesses a high degree of crystallinity on average. In the next nn shells, and in the case of all edges, the detected discrepancies can be attributed to the large error in the estimation of structural parameters (interatomic distances and DW factors) in such distant nn shells.

The atomic percentage of the Hf, Zr, and Ti atoms calculated by the EXAFS analysis verifies the stoichiometry expected by the EDS spectra, i.e., Ti_0.28_Zr_0.42_Hf_0.3_NiSn_0.48_Sb_0.02_. Indeed, the analysis in all edges revealed that the atomic percentage (at %) of Hf ranges between 29 (±5)–35 (±4) and of Zr lies between 42 (±5) and 47 at% (± 4); these values are practically equal—within the error bar—to the respective values predicted by EDS. The determined Hf and Zr content indicates that a degree of chemical inhomogeneity is expected within the material; however, it lies within the theoretical contents overall, taking into account the sample volume probed by EXAFS and the error in estimations. Thus, it is concluded that incorporating Hf in a (Ti,Zr)NiSnSb HH solid solution and doping with antimony as a next step yields a compound with a high crystalline order in the mid- and long-range order. On the other hand, the variations revealed by both EXAFS and TEM analysis imply the formation of a considerable amount of lattice point defects, such as interstitials and substitutional ions, which can significantly affect the TE properties. This finding further supports the direct relation between the nanostructure and thermal properties of HH alloys. More significantly, the incorporation of antimony into the lattice led to the enhancement of electrical conductivity [22], a finding that can be attributed to the material’s high crystalline order revealed by the EXAFS analysis results. The moderate increase in thermal conductivity could be assigned to the varied nature of point defects and interstitials among particles of various sizes in the material [22].

## 4. Conclusions

EXAFS spectroscopy is implemented for the detection of lattice disorder in a mechanically prepared Sb-doped (Zr,Hf,Ti)NiSn alloy in an effort to detect atomic-scale deviations, site disorder, and vacancies, which can directly impair the material’s thermoelectric properties. The analysis accuracy was further enhanced by the combination of SAD, (HR)TEM, and EDS with the EXAFS analysis results. It is revealed that Zr and Hf atoms preferentially substitute Ti atoms in the lattice; yet, static disorder, as determined by lattice expansion due to the larger atomic sizes of the Hf and Zr atoms, is not present. Indeed, as the EXAFS analysis results demonstrate, the occupation of octahedral sites by the dopants causes limited disturbance in the lattice crystal structure, hence preserving the high crystalline state of the particles. However, the introduction of a considerable density of point defects by Zr-Hf alloying and Sb doping, especially of substitutional nature, can predominantly result in a reduction in thermal conductivity and, most importantly, in a significant increase in electrical conductivity. The detected increased crystalline order in the mid- and long-range order and simultaneous point defect population of the HH alloy can be justified on the basis that, in the Sb-doped (Zr,Hf,Ti)NiSn alloy, alloying and doping resulted in a figure of merit maximum, as a consequence of optimized electrical and thermal conductivities, in a reverse manner.

## Figures and Tables

**Table 1:** Measurements of the lattice constant in the Sb-doped (Zr,Hf,Ti)NiSn sample, as derived by the SAD pattern (inset of Figure 2a). Spots in parentheses denote kinematically forbidden reflections, existing due to the alloying.

SAD Spot	111	200	(210)	220	(321)	333
*d*-spacing (Å)	3.501	3.022	2.696	2.133	1.611	1.174
*a* (Å)	6.064	6.045	6.029	6.032	6.027	6.102

**Table 2:** Quantitative EDS results for the Sb-doped (Zr,Hf,Ti)NiSn sample. The upper part of the table lists the absolute average values of each element content, whereas the lower part shows their ratios, normalized to the Ni content. The (nominal) sums of each of the distinct unit cell sites (C: Sn + Sb, N: Ti + Zr + Hf and T: Ni) are also included for comparison reasons.

Elements (at%)	Ti *K*	Zr *L*	Hf *M*	Ti + Zr + Hf	Ni *K*	Sn *L*	Sb *L*	Sn + Sb
Average	9.6	10.4	13.8	33.8	34	28	4	32
Nominal	9.33	14	10	33.3	33.3	32.7	0.7	33.4
Difference (%)	2.9	−25.7	38	1.5	2.1	−14.4	N/A	−4.2
Average (*Ni-norm.*)	0.28	0.31	0.41	0.99	1	0.82	0.18	0.94
Nominal (*Ni-norm.*)	0.28	0.42	0.3	1	1	0.98	0.02	1
Difference (%)	0	−26.2	36.7	−1	0	−16.3	N/A	−6

**Table 3:** EXAFS analysis results of all spectra recorded at the *K*-edges of Sn, Ni, and Zr and *L*_3_-edge of Hf. R denotes interatomic distances, nn is the abbreviation of nearest neighbor shell, and DW is the value of the Debye Wallet factor. x and y refer to the atomic percentage of the Hf and Zr atoms according to the suggested stoichiometry (Zr_x_Hf_y_Ti_z_)NiSn_0.98_Sb_0.02_.

		Sn-*K* Edge	Ni-*K* Edge	Zr-*K* Edge	Hf-*L*_3_ Edge
	at % Zr Atoms at % Hf Atoms	x = 45 ± 2 y = 30 ± 2	x = 47 ± 4 y = 35 ± 4	x = 42 ± 5 y = 29 ± 5	x = 42 ± 3 y = 28 ± 3
**1st nn shell**	**Atom type**	Ni	Sn	Ni	Ni
	R (Å) (±0.01)	2.61	2.60	2.64	2.61
	DW (×10^−3^Å^2^)	6.3	6.5	5.6	6.3
**2nd nn shell**	**Atom type**	Zr	Zr	Sn	Sn
	R (Å) (±0.02)	3.05	2.65	3.05	3.02
	DW (×10^−3^Å^2^)	4.2	6.1	5.1	4.5
	**Atom type**	Hf	Hf		
	R (Å) (±0.03)	3.04	2.65		
	DW (×10^−3^Å^2^)	4.5	13.0		
	**Atom type**	Ti	Ti		
	R (Å) (±0.04)	3.05	2.60		
	DW (×10^−3^Å^2^)	4.5	4.2		
**3rd nn shell**	**Atom type**	Sn	Ni	Zr	Zr
	R (Å) (±0.05)	4.29	4.29	4.27	4.29
	DW (×10^−3^Å^2^)	9.2	9.0	3.8	1.2
	**Atom type**			Hf	Hf
	R (Å) (±0.06)			4.28	4.27
	DW (×10^−3^Å^2^)			4.2	1.5
	**Atom type**			Ti	Ti
	R (Å) (±0.07)			4.27	4.30
	DW (×10^−3^Å^2^)			2.9	2.2
**4th nn shell**	**Atom type**	Ni	Zr	Ni	Ni
	R (Å) (±0.08)	5.04	4.98	5.04	5.00
	DW (×10^−3^Å^2^)	18.0	5.5	4.7	7.6
	**Atom type**		Hf		
	R (Å) (±0.09)		5.03		
	DW (×10^−3^Å^2^)		1.9		
	**Atom type**		Ti		
	R (Å) (±0.10)		4.94		
	DW (×10^−3^Å^2^)		3.3		
**5th nn shell**	**Atom type**	Zr	Sn	Sn	Sn
	R (Å) (±0.11)	5.18	4.97	5.04	5.06
	DW (×10^−3^Å^2^)	1.8	12.5	9.2	18.1
	**Atom type**	Hf			
	R (Å) (±0.12)	5.20			
	DW (×10^−3^Å^2^)	4.5			
	**Atom type**	Ti			
	R (Å) (±0.13)	5.17			
	DW (×10^−3^Å^−2^)	1.9			
**6th nn shell**	**Atom type**	Sn	Ni	Zr	Zr
	R (Å) (±0.14)	5.98	6.01	3.09	6.01
	DW (×10^−3^Å^2^)	6.6	9.9	29.2	6.2
	**Atom type**			Hf	Hf
	R (Å) (±0.15)			5.86	6.17
	DW (×10^−3^Å^−2^)			2.6	4.9
	**Atom type**			Ti	Ti
	R (Å) (±0.16)			5.81	5.90
	DW (×10^−3^Å^2^)			4.4	17.2
**7th nn shell**	**Atom type**	Ni		Ni	Ni
	R (Å) (±0.17)	6.63		6.64	6.55
	DW (×10^−3^Å^2^)	9.5		12.0	7.6
**8th nn shell**	**Atom type**	Zr		Sn	Sn
	R (Å) (±0.18)	6.66		6.80	6.80
	DW (×10^−3^Å^2^)	8.7		11.0	9.7
	**Atom type**	Hf			
	R (Å) (±0.19)	6.67			
	DW (×10^−3^Å^2^)	8.7			
	**Atom type**	Ti			
	R (Å) (±0.20)	6.67			
	DW (×10^−3^Å^2^)	9.3			
**9th nn shell**	**Atom type**	Sn		Zr	Zr
	R (Å) (±0.21)	7.44		7.26	7.39
	DW (×10^−3^Å^2^)	9.3		22.0	6.1
	**Atom type**			Hf	Hf
	R (Å) (±0.22)			7.27	7.39
	DW (×10^−3^Å^2^)			3.8	4.9
	**Atom type**			Ti	Ti
	R (Å) (±0.23)			7.45	7.40
	DW (×10^−3^Å^2^)			5.9	17.0
**10th nn shell**	**Atom type**	Ni		Ni	Ni
	R (Å) (±0.24)	8.0		7.63	7.58
	DW (×10^−3^Å^2^)	14.1		4.8	13.1

## Data Availability

The raw data supporting the conclusions of this article will be made available by the authors on request.
